# Molecular typing and characterization of nasal carriage and community-onset infection methicillin-susceptible S*taphylococcus aureus* isolates in two Taiwan medical centers

**DOI:** 10.1186/1471-2334-12-343

**Published:** 2012-12-10

**Authors:** Feng-Jui Chen, Leung-Kei Kristopher Siu, Jung-Chung Lin, Chen-Her Wang, Po-Liang Lu

**Affiliations:** 1 Division of Infectious Diseases, National Health Research Institutes, Zhunan, Taiwan; 2 Graduate Institute of Basic Medical Science, China Medical University, Taichung, Taiwan; 3 Department of Internal Medicine, Division of Infectious Diseases and Tropical Medicine, Tri-Service General Hospital, Taipei, Taiwan; 4 Department of Internal Medicine, Kaohsiung Medical University Hospital, Kaohsiung, Taiwan; 5 School of Medicine, College of Medicine, Kaohsiung Medical University, Kaohsiung, Taiwan

**Keywords:** Nasal carriage, Community onset, Infection, MSSA, *Staphylococcus aureus*, Lineage, Pulsed-field gel electrophoresis, Multi-locus sequence typing, *Spa* typing

## Abstract

**Background:**

Compared to methicillin-resistant *Staphylococcus aureus* (MRSA), characteristics of nasal carriage and community-onset infection methicillin-susceptible *S. aureus* (MSSA) are less well known. No characteristics of MSSA in Taiwan have been reported previously.

**Methods:**

We analyzed 100 nasal carriage and 34 community-onset infection MSSA isolates by pulsed-field gel electrophoresis (PFGE), *spa* typing, multi-locus sequence typing, *agr* typing, virulence gene detection, growth rate measurement, and antimicrobial susceptibility.

**Results:**

In PFGE analysis, most (68%) infection isolates could be grouped in one major cluster using a 70% similarity cutoff. In contrast, only 17% of nasal carriage isolates belonged to this cluster. A similar classification was obtained using Based Upon Repeat Pattern analysis of *spa* types. The MSSA infection isolates cluster was closely related to the virulent clones of clonal complex 1 (CC1), which includes strains MW2 (USA400) and MSSA476. ST188 of CC1 was the predominant clone detected for community-onset MSSA infections. The only common ST type for MSSA and MRSA in Taiwan was ST59, the community-associated MRSA clone. It is likely, therefore, that MRSA originated from MSSA clones through SCC*mec* transfer. Compared to nasal carriage isolates, infection isolates less frequently possessed *egc*, *tst* and *hlg* genes, were more commonly susceptible to erythromycin (91% vs. 54%), and had shorter mean doubling times (38 min vs. 55 min).

**Conclusions:**

The clonal lineages of MSSA nasal carriage and infection isolates differed in our sample of Taiwan isolates. Most community-onset MSSA infections resulted from relatively few clonal lineages. Nasal carriage isolates more frequently possessed the *egc*, *tst* and *hlg* genes, were more resistant to erythromycin, and grew more slowly.

## Background

*Staphylococcus aureus* can asymptomatically colonize people in the community and in the healthcare setting. *S. aureus* is also responsible for a wide spectrum of illnesses, ranging from superficial infection of the skin and soft tissue to life-threatening septicemia, osteomyelitis, endocarditis, and toxic shock syndrome [1]. A few clonal lineages have also been observed among epidemic methicillin-resistant *S. aureus* (MRSA) isolates [2]. Although successful lineages of epidemic MRSA clones may have an adaptive advantage because of antibiotic resistance, virulence, and gene regulation, the exact mechanism by which these clones successfully circulate in community and healthcare facilities has not been fully elucidated. A previous study found that most MRSA clones arose from successful epidemic methicillin-susceptible *S. aureus* (MSSA) clones [2]. It would be interesting to have further studies to investigate if successful MSSA clones could be used to predict prevalent strains of MSSA or MRSA infection.

It has been shown that nasal carriage of *S. aureus* is a major risk factor for subsequent development of community-associated and nosocomial infections [3,4]. Although the relationship between bacterial virulence determinants and invasive disease has been explored by comparing nasal carriage and disease isolates [5], most studied isolates were obtained from the healthcare environment. However, *S. aureus* isolates obtained from hospital patients may represent nosocomial transmission, and could therefore confound understanding of the intrinsic pathogenesis of specific *S. aureus* strains. The aim of the present study was to identify differences in lineages, virulence gene prevalence, growth rates and antimicrobial susceptibility between nasal carriage and community-onset infection MSSA.

## Methods

### Bacterial isolates

Thirty-four community-onset infection MSSA isolates were obtained from patients at two Taiwan medical centers (Tri-Service General Hospital and Kaohsiung Medical University Hospital) during a four-month collection period in 2006. A total of 100 nasal carriage MSSA isolates were available from our previous study [6]. The 34 community-onset infections were classified as community-associated infection or healthcare-associated community-onset infection according to the definition of Klevens et al. [7]. The criteria used to classify community-associated infection and healthcare-associated community-onset infection are as follows:

1. Community-associated infection: No permanent indwelling catheters or medical devices that pass through the skin into the body and no medical history in the past year of hospitalization, admission to nursing home or nursing facility or hospice. Isolates from the specimens should be obtained within 48 hours after admission to hospital.

2. Healthcare-associated community-onset infection: Patient with medical history of hospitalization, admission to nursing home or nursing facility or hospice in the past year. Isolates from the specimens should also be obtained within 48 hours after admission to hospital.

### Pulsed-field gel electrophoresis (PFGE)

Staphylococcal genomic DNA typing was performed using PFGE by *Sma*I digestion, as previously described [8]. PFGE clusters were assigned to isolate clusters having 80% or higher similarity from the dendrogram based on Dice’s coefficients [8]; genetic relatedness was also assessed by visual inspection of fragment differences [9]. Analysis of isolate similarity was also performed, using a cut-off value of 70%.

### *spa* types, multi-locus sequence typing (MLST), *agr* specificity groups, and virulence genes

We used PCR to molecularly characterize isolates by *spa* type, multi-locus sequence typing (MLST), *agr* specificity group, and virulence genes. *S. aureus* DNA was extracted using a DNeasy Tissue Kit (Qiagen Inc., Valencia, USA), following the manufacturer’s instructions with a slight modification. An additional step was added just prior to proteinase K digestion: incubation with 5 μL of 5 mg/mL lysostaphin at 37°C for 30 min to 1 h until the cell suspension became clear.

*spa* typing and the Based Upon Repeat Pattern (BURP) algorithm were performed using Ridom StaphType software (version 1.5; Ridom GmbH, Würzburg, Germany). Two clustering parameters were applied to BURP clusters, *spa*-clonal complexes (CCs), using dialog: “exclude *spa* types that are shorter than 5 repeats” and “*spa* types are clustered if cost is less or equal 4” [10,11]. The cost “6” parameter was also used as discussed by Strommenger et al. [12]. The cost matrix for distances of strains between *spa* types was also produced using the StaphType software, and the phylogenetic tree was conducted using MEGA version 5 by the neighbor-joining method [13]. The *spa* repeat region was amplified and sequenced using primers spa-1113f and spa-1514r, according to the manufacturer's instructions.

MLST was performed as described previously on selected isolates of major PFGE clusters and all infection isolates [14]. Sequence type (ST) was assigned based on sequence allelic profiles using the MLST database website (http://www.mlst.net) [14].

*agr* specificity groups were determined by multiplex PCR as described by Lina et al. [15]. Non-typable strains were determined by *agrD* sequencing, which encodes the autoinducing peptide (AIP) precursor [16].

Virulence toxin genes for *sea-e*, *seg-j*, *sem-o*, *tst*, *eta*, *etb*, *lukElukD*, *hla*, *hlb*, *hld*, *hlg*, and *hlg-2* were detected as described by Jarraud et al. [17]. Other bacterial adhesion genes, including *fnbA*, *cna*, *sdrC*, *sdrD*, *sdrE*, *bbp*, and *icaA*, were detected as described by Peacock et al. [5]. Sequences specific for Panton-Valentine leukocidin (PVL) genes (*lukS-lukF*) were detected as described previously [18].

### Bacterial growth rate

A colorimetric microplate assay was used to assess doubling time of representative clonal lineage strains [19,20]. Isolates were grown overnight in Tryptic Soy Broth (TSB), diluted 100-fold, then grown for an additional 2 h. Samples were washed twice with phosphate-buffered saline (PBS), then the pellet was re-suspended in TSB to a concentration of approximately 10^9^ CFU/mL. Serial ten-fold dilutions were made to 10^8^ to 10^4^ CFU/mL with TSB. One hundred microliters of each dilution were plated in triplicate on microplates, and TSB without cells served as a control. Then 20 μL of CellTiter-Blue® reagent (Promega Corp., Madison, USA) were added to each well, and the cells were incubated at 37°C. The absorbance was recorded every 30 min until each dilution of cells reached maximum absorbance (OD at 570 nm and 600 nm as reference wavelengths). The doubling time was calculated as previously described [19].

### Antibiotic susceptibility testing

Susceptibilities were determined based on minimum inhibitory concentrations obtained from broth micro-dilution following the guidelines of the Clinical and Laboratory Standards Institute [21], using Sensititre custom-designed plates (Trek Diagnostics, West Essex, England).

### Clinical data and statistical analysis

The study was performed with approval from the Institutional Review Board of Kaohsiung Medical University Hospital (KMUH-IRB-990139). Data for cases with MSSA nasal carriage were obtained following written informed consent [6]. Differences in frequencies and proportions were tested using the chi-square (χ^2^) test with Yates' correction or Fisher's exact test, as appropriate. A *P* value of less than 0.05 was considered significant. To compare virulence gene profiles, factors associated with infection at *P* values less than 0.01 were further studied using a logistical regression model to calculate the odd ratios (ORs) and 99% confidence intervals (CIs).

## Results

### Genetic profiling of nasal carriage and community-onset infection isolates

**Figure 1:**
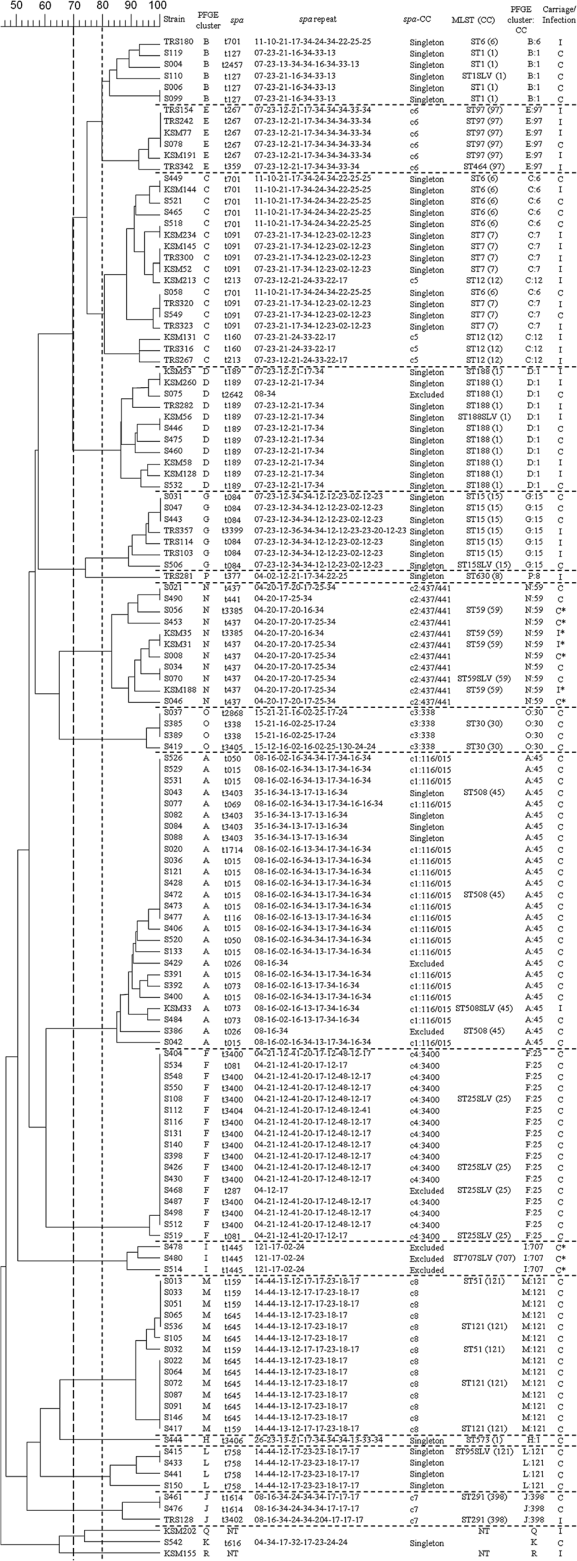
**PFGE dendrogram with molecular characterization for 100 nasal carriage and 34 infection MSSA isolates.** PFGE cluster was assigned to isolates having 80% or greater similarity from the dendrograms (A similarity cut-off of 70% was also presented.). Multi-locus sequence typing (MLST), results in sequence type (ST); clonal complexes (CC); * Panton-Valentine leukocidin (PVL)-positive.

Molecular typing results, including PFGE, *spa* typing and MLST are presented in Figure 1. A total of 18 distinct PFGE clusters were found among 134 isolates, based on an 80% similarity cut-off value (approximately equivalent to less than or equal to six PFGE fragment differences). Thirty-nine *spa* types, including 7 new ones, were identified; two isolates were non-typable. Grouping by BURP, these *spa* types were clustered into 8 *spa*-CCs and 12 singletons. Four of eight *spa*-CCs had designated group founders: *spa*-CC c1:t116/t015, c2:t437/t441, c3:t338, and c4:t3400. Seven isolates with fewer than five repeats were excluded from clustering because their *spa* types may provide unreliable evolutionary information [11]. Although our results showed a high concordance among *spa* typing, MLST and PFGE, PFGE cluster C contained many *spa* types and STs. Four *spa* types (t701, t091, t213 and t160) and three STs (ST6, ST7 and ST12) were found in PFGE cluster C. These STs were distinct from one another and belonged to different clonal complexes (CCs). Because some MLST STs may be grouped in specific PFGE clusters, clonal lineages were described according to the combination of PFGE cluster and CC (PFGE cluster:CC) in the study (Figure 1).

### *S. aureus* infections and clinical characteristics

The 34 community-onset infection MSSA isolates were classified as 24 community-associated isolates and 10 healthcare-associated community-onset isolates (Table 1). There were no significant differences between clinical manifestations caused by community-associated and healthcare-associated community-onset isolates. There were no associations between clonal lineages and disease manifestations. All STs of healthcare-associated community-onset isolates were also identified in community-associated isolates (Table 1). Therefore, all 34 MSSA infection isolates were grouped together for analysis.

**Table 1:** **Clinical characteristics of patients with community-associated and healthcare-associated community-onset *S. aureus *infection**

**Category (No.)**	**PFGE cluster:CC**^**#**^	MLST*	**Diagnosis**^**#**^	
			**Superficial infection**	**Deep infection**
Community-associated infection (24)	A:45 (1)	ST508	Cellulitis	
	B:6 (1)	ST6		Osteomyelitis
	C:7 (3)	ST7	Cellulitis (1)	Pneumonia (1), spondylitis (1)
	C:12 (3)	ST12		Necrotizing mediastinitis (1), acute cholecystitis (1), spondylitis (1)
	D:1 (5)	ST188	Urinary tract infection (1)	Spondylitis (1), paraspinal abscess (1), septic arthritis (1), pneumonia (1)
	E:97 (3)	ST97	Cellulitis (1)	Septic arthritis (1), endocarditis (1)
	G:15 (2)	ST15	Cellulitis (2)	
	J:398 (1)	ST291		Septic arthritis
	N:59 (2)	ST59	Cellulitis (2)	
	P:8 (1)	ST630		Epiglotitis with septicemia
	Q (1)	NT		Pneumonia
	R (1)	NT		Pneumonia
Healthcare-associated community-onset infection (10)	C:6 (1)	ST6		Pneumonia
	C:7 (3)	ST7	Wound (2)	CNS infection (1)
	C:12 (1)	ST12	Wound	
	D:1 (2)	ST188	Epidermal cyst (1), wound (1)	
	E:97 (1)	ST97		Septic arthritis
	G:15 (1)	ST15		Bacteremia
	N:59 (1)	ST59	Wound	

*PFGE*: pulsed-field gel electrophoresis.

*MLST*: multi-locus sequence typing.

*NT*: non-typable.

* representative STs.

^#^ the number in parenthesis represents the number of isolates.

### Distribution of clonal lineages among nasal carriage and infection isolates

Although PFGE cluster C contained three different CCs (CC6, CC7 and CC12), isolates from this PFGE cluster were significantly more prevalent in infection than carriage isolates (32% vs. 6%, *P* < 0.01). An intriguing observation was that, using a 70% similarity cut-off value for cluster analysis (approximately equivalent to less than or equal to eight PFGE fragment differences) resulted in most infection isolates being grouped into one major cluster (including PFGE clusters B, C, D and E), with 68% of infection isolates and 17% of carriage isolates (*P* < 0.01). Carriage isolates were more diverse than infection isolates (Figure 1). The major infection isolates cluster contained several clonal lineages, including B:1, B:6, C:6, C:7, C:12, D:1 and E:97. STs of these infection isolates were ST1, ST6, ST6, ST7, ST12, ST188, and ST97, respectively. These ST isolates belonged to five CCs: CC1, CC6, CC7, CC12 and CC97. These data implicate specific MSSA clones as causing infectious diseases when clonal lineage was defined by PFGE and MLST typing. ST188 of CC1 was the predominant clone for community-onset MSSA infections. ST59 comprised 8.2% of all MSSA isolates but did not belong to the major infection isolates cluster.

**Figure 2:**
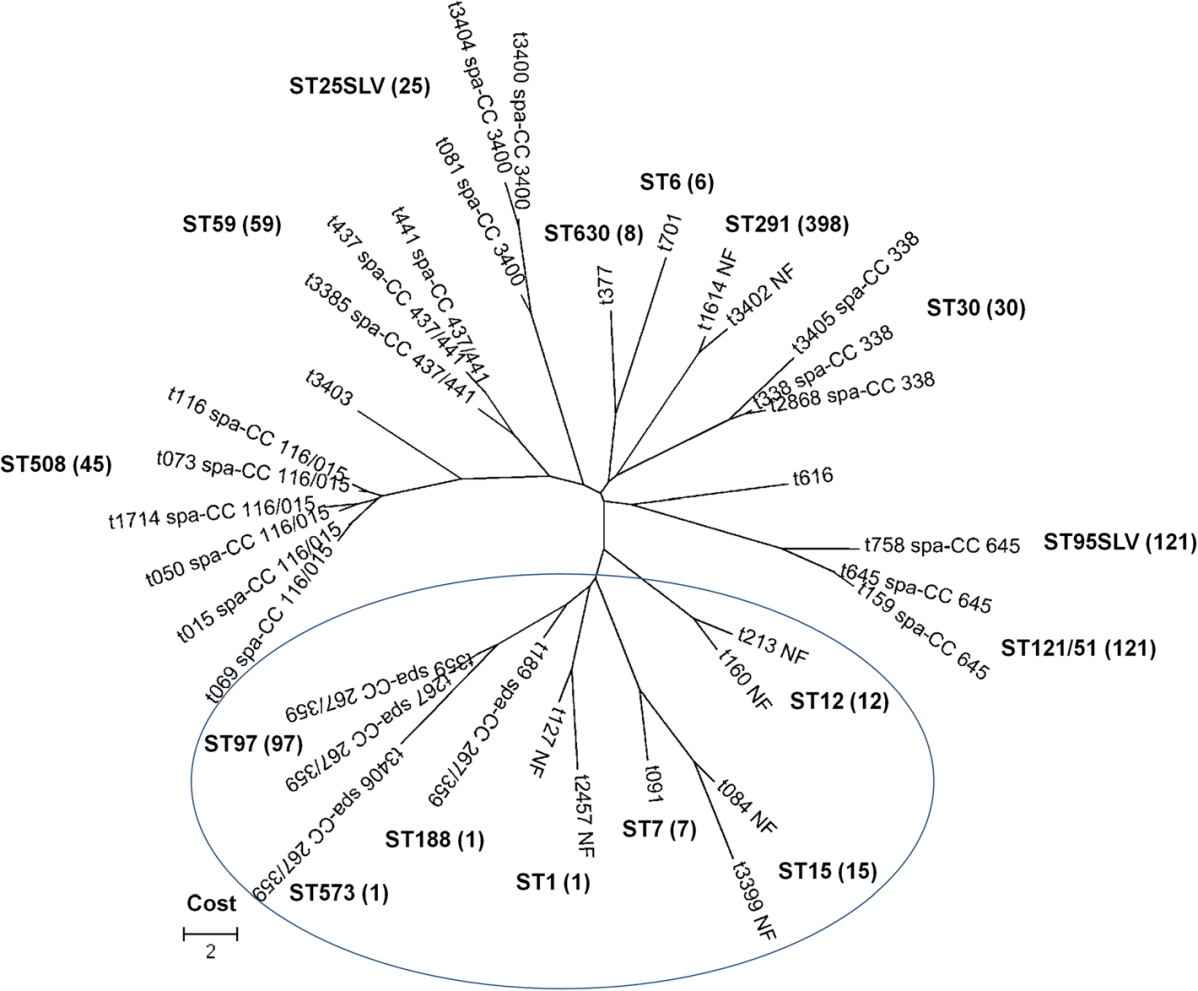
**Phylogenetic tree of the *spa *types was constructed by using MEGA version 5.** The tree was produced based on the cost matrix for the distances of *spa* types. The cost “6” parameter for the BURP clustering was used in this figure. The circle indicates the major infection PFGE cluster. *spa*-CCs and the represented ST (CC) are indicated. NF (no founder) indicates clusters without group founders.

BURP analysis of *spa* types showed a similar phenomenon to the PFGE clusters result. In an attempt to cluster more *spa* types in *spa*-CCs, we tried to adjust the cost to 6 for clustering more *spa* types in the BURP group; only *spa*-CC 267/359 could be grouped together among the major infection isolates. However, as indicated by the circle in Figure 2, the major infection PFGE cluster had closely related *spa* types by BURP analysis, with most infection PFGE clusters grouped together (except CC6).

### *agr* specificity groups and virulence gene profiles

*agr* group I was the most common *agr* type for both nasal carriage (65%) and infection (74%) isolates, followed by *agr* group II for infection isolates (21%) and *agr* group IV for nasal carriage isolates (18%).

Among 30 virulence genes, only the LukE-LukD leukotoxin (*lukE-lukD*) and gamma variant hemolysin (*hlg-2*) genes were significantly more prevalent in infection isolates than in nasal carriage isolates (Table 2). In contrast, seven virulence genes were significantly more common in nasal carriage isolates than in infection isolates. Five of these seven virulence genes were staphylococcal enterotoxin genes (*seg, sei, sem, sen,* and *seo*), which reside at the enterotoxin gene cluster (*egc*) locus. Two other virulence genes found were *tst* and *hlg*, which encode toxic shock syndrome toxin 1 (TSST-1) and gamma-hemolysin, respectively. Although these were all community isolates, PVL toxin genes (*lukS-lukF*) were uncommonly detected in nasal carriage (7%) and infection (8.8%) isolates (*P*=0.727).

**Table 2:** **Comparison of virulence gene profiles among *S. aureus *nasal carriage and infection isolates**

**Virulence gene**	**Carriage**		**Infection**				
	**N**	**(%)**	**N**	**(%)**	**OR**	**99% CI**	***P***
*fnbA*	91	91	33	97.1	3.264	0.206-51.833	0.271
*cna*	58	58	14	41.2	0.507	0.179-1.432	0.092
*sdrC*	100	100	34	100			
*sdrD*	59	59	20	58.8	0.993	0.351-2.806	0.986
*sdrE*	72	72	24	70.6	0.933	0.303-2.879	0.875
*bbp*	23	23	2	5.9	0.209	0.029-1.507	0.041
*icaA*	97	97	34	100			
*sea*	14	14	6	17.6	1.316	0.332-5.212	0.607
*seb*	29	29	6	17.6	0.525	0.144-1.907	0.198
*sec*	26	26	4	11.8	0.379	0.085-1.686	0.094
*sed*	0	0	0	0			
*see*	0	0	0	0			
***seg***	**65**	**65**	**2**	**5.9**	**0.034**	**0.005**-**0.237**	**<0.001**
*seh*	37	37	12	35.3	0.929	0.319-2.700	0.858
***sei***	**64**	**64**	**6**	**17.6**	**0.121**	**0.034**-**0.432**	**<0.001**
*sej*	0	0	0	0			
***sem***	**66**	**66**	**2**	**5.9**	**0.032**	**0.005**-**0.227**	**<0.001**
***sen***	**67**	**67**	**1**	**2.9**	**0.015**	**0.001**-**0.216**	**<0.001**
***seo***	**66**	**66**	**2**	**5.9**	**0.032**	**0.005**-**0.227**	**<0.001**
***tst***	**31**	**31**	**1**	**2.9**	**0.067**	**0.005**-**0.977**	**0.009**
*eta*	20	20	1	2.9	0.121	0.008-1.790	0.044
*etb*	12	12	0	0			
***lukE*-*lukD***	**69**	**69**	**32**	**94.1**	**7.188**	**1.014**-**50.947**	**0.009**
*lukS-lukF*	7	7	3	8.8	1.286	0.201-8.226	0.727
*hla*	97	97	33	97.1	1.021	0.050-20.899	0.986
*hlb*	31	31	8	23.5	0.685	0.210-2.231	0.409
*hld*	100	100	34	100			
***hlg***	**34**	**34**	**2**	**5.9**	**0.121**	**0.017**-**0.857**	**0.005**
***hlg2***	**63**	**63**	**30**	**88.2**	**4.405**	**1.012**-**19.181**	**0.009**

### Bacterial growth rate

Doubling time was calculated to determine bacterial growth rates for all infection isolates and representative nasal carriage isolates (one was randomly selected from each nasal carriage clonal lineage). The mean doubling times for nasal carriage isolates were significantly higher compared to infection isolates (55.3 ± 8.1 vs. 37.9 ± 1.2 minutes, *P* < 0.01). Of 30 virulence genes, only *egc* enterotoxin genes were associated with significantly higher growth rates (*P* < 0.01).

### Antibiotic resistance profiles

Antimicrobial resistance profiles were similar among the two groups of isolates. However, erythromycin resistance was found for 46% of nasal carriage isolates versus only 9% of infection isolates (*P* < 0.01) (Table 3).

**Table 3:** **Comparison of resistance rates (%) for *S. aureus *nasal carriage and infection isolates**

**Antimicrobial agent**	**Carriage (n=100)**	**Infection (n=34)**	***P***
	**N (%)**	**N (%)**	
Chloramphenicol	10 (10%)	3 (9%)	1
Ciprofloxacin	0	0	
Clindamycin	13 (13%)	3 (9%)	0.56
Erythromycin	46 (46%)	3 (9%)	**<0.01**
Gentamicin	0	2 (6%)	0.07
Linezolid	0	0	
Oxacillin	0	0	
Penicillin	92 (92%)	31 (91%)	0.73
QDA	0	0	
Rifampin	1 (1%)	0	1
SXT	0	0	
Teicoplanin	0	0	
Tetracycline	36 (36%)	14 (41%)	0.92
Vancomycin	0	0	

*QDA*: Quinupristin/dalfopristin.

*SXT*: Trimethoprim/sulfamethoxazole.

## Discussion

Compared to previous MRSA clone studies in Taiwan, which showed only four major infection isolate clones (ST239, ST59, ST241 and ST5) [22,23], our study revealed more heterogeneous MSSA lineages. The major PFGE cluster of MSSA infection isolates included ST1, ST6, ST7, ST12, ST188, and ST97. However, these STs have not been reported in MRSA isolates in Taiwan [22,23], indicating that most MSSA lineages have a different genetic background compared to MRSA lineages. Additionally, these MSSA STs differ from the most prevalent *S. aureus* STs worldwide [24]. Interestingly, the only ST common to MSSA and MRSA [22,23] in Taiwan is ST59, the representative community-associated MRSA clone [25]. Our findings are consistent with those from several other countries, suggesting that MRSA probably originated from MSSA clones through SCC*mec* transfer [26]. These results, plus findings from a world-wide MRSA collection study, point to MRSA evolution in relatively few lineages [2], which allowed some successful clones to cause disease or spread. Although not all published studies have shown clonal differences between nasal carriage and infection isolates [27], our study revealed clonal differences in MSSA infection and nasal carriage isolates. MSSA infection isolates’ distinctive lineages and erythromycin susceptibility, as well as faster growth rates, support the clones’ uniqueness to cause community-onset MSSA infection.

Our finding that STs and clinical manifestations did not differ between community-associated and healthcare-associated community-onset isolates is consistent with Peacock et al.’s findings [5]. That team found little difference between strains associated with disease in both community and hospital settings, and suggested that bacterial factors play a role in determining invasive disease. In the present study, PFGE cluster D: CC1 (ST188) was the predominant clone in the community-onset MSSA infections. Our previous study also showed that this clone predominated among MSSA isolates from inpatients in a 2002 collection from four hospitals located in the north, middle, south, and east regions of Taiwan [16]. These results indicated that the epidemiology of MSSA infection isolates did not change much between these two periods, with no difference noted in either community or hospital settings.

Using PFGE or BURP analysis of *spa* types, we identified a few lineages likely to cause infection. Most of these representative CCs have been described previously as “group violations”, with the highest degree of misclassification between BURP groups and clonal lineages [12,28]. However, these isolates may have clinical relevance because of their close relationship with severe community-associated infection strains in humans (MSSA476 and MW2 (USA400)) and ocular conjunctival infection in rabbits (UMCR1). All of these isolates belong to CC1 [29-31], and their high virulence has been shown in animal models [30,32].

To clarify the difference between the nasal carriage isolates and infection isolates, we also compared the virulence gene profiles and growth rates among isolates. Infection isolates grew faster than carriage isolates. This difference suggests that infection isolates have better proliferation ability to outcompete other clones in the host. Additionally, the LukE-LukD leukotoxin (*lukE-lukD*) and gamma variant hemolysin (*hlg-2*) genes were more prevalent in infection isolates. Although seven virulence genes (*egc* enterotoxin genes, *tst* and *hlg*) were more prevalent in nasal carriage isolates, only isolates with *egc* enterotoxin genes were associated with slower growth rates (*P* < 0.01). It is possible that *egc* enterotoxin genes pose a fitness burden to the strains, resulting in slowed growth rates. The *egc* enterotoxin genes have been observed to have low-level production and weak virulence effects [33-35]. Our results reveal an epidemiologic relationship between *egc* enterotoxin genes and growth rate; however, the cause-effect relationship cannot be established in this study. It has been reported that virulence gene profiles are strongly linked with *S. aureus* clonal lineages [17]. Our nasal carriage isolates have uncommon STs, with most possessing the *egc* enterotoxin genes. Previous studies also showed such an epidemiological phenomenon in nasal carriage isolates [5,36]. However, the role of the *egc* enterotoxin genes in nasal carriage isolates is not yet understood. The reason why our nasal carriage isolates have higher erythromycin resistance rates compared to community-onset infection isolates is also unclear.

Although the PVL genes have been considered a marker for community-acquired MRSA [37], we did not find that PVL presence was associated with community-onset MSSA infections. Goering et al. also failed to find this PVL association in a recent study [24].

*agr*, a global virulence gene regulator of *S. aureus*, is strongly linked with clonal lineages and some disease syndromes [17]. *agr* group I isolates were prevalent in our study, as in a previous study [38]. A similar distribution of *agr* group I was observed among nasal carriage (65%) and infection isolates (74%). *agr* types and PVL toxin genes were not useful in predicting infection MSSA isolates in the community setting.

Though only 34 infection isolates were analyzed, these isolates originated from two medical centers over a four-month period. The low MSSA isolate numbers result from a high rate of methicillin resistance among *S. aureus* in Taiwan during the study period [6,22]. Also, only community-onset MSSA isolates were collected for the present study.

## Conclusions

Our study presents genetic differences between nasal carriage and infection MSSA isolates from a community setting in Taiwan. The major MSSA infection isolates collected were closely related to the virulent clonal complex CC1. In addition to the difference in clonal lineages between nasal carriage and infection isolates, nasal carriage isolates more frequently possessed *egc*, *tst* and *hlg* genes, were more resistant to erythromycin, and had slower growth rates.

## Competing interests

The authors declare that no competing interests exist.

## Authors’ contributions

FJC and PLL designed the study. JCL provided advice regarding clinical aspects of the study. FJC and CHW performed laboratory work. PLL, LKS, and FJC prepared the manuscript. All authors read and approved the final version of the manuscript.

## Pre-publication history

The pre-publication history for this paper can be accessed here:

http://www.biomedcentral.com/1471-2334/12/343/prepub
